# A novel approach to non-segmented flow analysis: Part 3. Nitrate, nitrite and ammonium in waters

**DOI:** 10.1155/S1463924688000410

**Published:** 1988

**Authors:** D. J. Malcolme-Lawes, C. Pasquini

**Affiliations:** Centre for Research in Analytical Chemistry and Instrumentation King's College London Strand London WC2R 2LS UK

## Abstract

A high-performance continuous-flow analyser is used for the analysis of nitrate, nitrite and ammonium ion in potable waters. The results demonstrate that (1) the analyser allows the sequential determination of a number of analytes without requiring modification to the manifold; (2) the use of programmed slicing of the reaction mixture allows a wide range of analyte concentrations to be handled; and (3) that the sensitivity achieved compares favourably with the best available from conventional flow-injection analysis. The limits of detection were found to be 5 ppb for NH_4_^+^, 30 ppb for NO_3_^-^, and 4 ppb for NO_2_^-^.


Journal of Automatic Chemistry Vol. 10, No. 4 (October–December 1988), pp. 192–197

### A novel approach to non-segmented flow analysis: Part 3. Nitrate, nitrite and ammonium in waters

**D. J. Malcolme-Lawes and C. Pasquini**

*Centre for Research in Analytical Chemistry and Instrumentation, King's College London, Strand, London WC2R 2LS*

*A high-performance continuous-flow analyser is used for the analysis of nitrate, nitrite and ammonium ion in potable waters. The results demonstrate that (1) the analyser allows the sequential determination of a number of analytes without requiring modification to the manifold; (2) the use of programmed slicing of the reaction mixture allows a wide range of analyte concentrations to be handled; and (3) that the sensitivity achieved compares favourably with the best available from conventional flow-injection analysis. The limits of detection were found to be 5 ppb for $NH_4^+$, 30 ppb for $NO_3^-$, and 4 ppb for $NO_2^-$.*

### Introduction

In two recent papers [1 and 2] a novel approach to non-segmented continuous-flow analysis was described in which a computer controlled valve switching system was used to permit the precise mixing of sample and reagents for selection reaction analysis. An improved version of the apparatus, which employed a computer-controlled colorimeter, provided excellent sample throughput and sensitivity, and several of the advantages offered by the new technique were discussed. In this paper one of those advantages is demonstrated, namely the ability to perform sequential analyses for different analytes without requiring modification to the apparatus. The technique is demonstrated using the analyses of nitrate, nitrite and ammonium ions in a variety of water samples.

Several reports [3–6] have described flow-injection analysis (FIA) systems for the determination of nitrate and nitrite ion in aqueous systems, either simultaneously or sequentially. In most cases Shinn's reaction is used for the determination of nitrite ion, and nitrate ion is determined in the same way after initial reduction to nitrite. This illustrates the point that such sequential determinations by FIA are generally possible only when the same reagent may be used in each determination, or when the same manifold could be employed for the determination of different analytes at the required levels. Thus most of the previous sequential $NO_2^-/NO_3^-$ determinations have been similar, as widely varying analyte concentrations normally involve changes to the manifold. Nitrate/nitrite determinations are generally performed under acidic conditions while the determination of ammonium ion is normally achieved using the Berthelot reaction [7] in alkaline media. By adopting a computer-controlled valve-switching approach we are able to change carriers and reagents with ease, so that sequential analyses involving different carriers and reagents may be performed, and to select different portions of the reaction product profile for different samples of analytes in order to dramatically improve the dynamic range for analyte determination.

### Experimental

The instrument [1,2] used in these experiments has remained much as described previously [2], although two additional valves have been incorporated in the manifold for the reasons discussed below, and the layout of the manifold is now as illustrated in figure 1. Furthermore, the variable wavelength visible absorbance monitor was fitted with a 2·5 cm path length flow cell for these experiments.

Carrier, reagent and sample flows were controlled using solenoid valves connected together as a manifold using 1/16th in od, 0·023 in id PTFE tubing. Valves 2 and 3 allowed a choice of principal carrier liquids, while valves 8–15 allow selected reagents, diluents or alternative carriers to enter the flow by replacing the principal carrier. By operating groups of valves alternately at a frequency of 20 Hz, it was possible to produce reagent mixtures in the manifold and this technique is particularly useful for reagent mixtures which would exhibit a short shelf-life, including those for the determination of $NH_4^+$ discussed below. The reagent or mixture selected for a particular determination was allowed to fill the manifold for a predetermined time period, referred to as the fill time ($t_f$), controlled by the computer and defined by a software procedure written for the determination. At this point the sample loop circuit (bound by valves 4 and 6) could be switched out of the manifold and filled with sample using the solenoid operated syringe pump. Excess liquid was expelled from the syringe pump through valve 1, so the pump could be operated repeatedly to fill any desired loop volume.

Once the sample loop had been loaded, the pathway between valve 15 and the reaction tube contained a carrier–reagent–sample–carrier (figure 2[*a*]) or a carrier–reagent–carrier–sample–carrier (figure 2[*b*]) pattern which depended on the magnitude of $t_f$ and was of precisely reproducible volumes. The first pattern gave maximum sensitivity, while the second could be used for an automatic in-line dilution type of procedure, in which only the highly dispersed portion of the sample zone mixed with reagent. Details of the volumes and reproducibility of these patterns have been discussed previously [2].

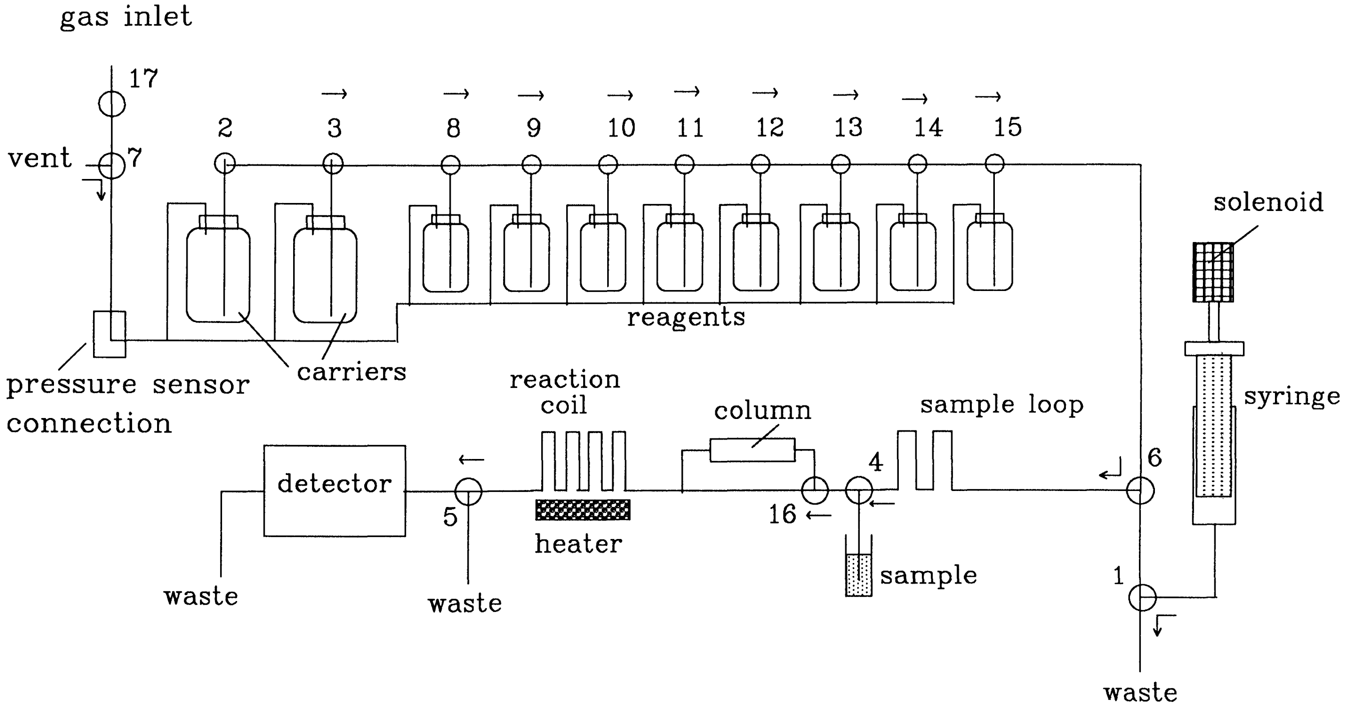

*Figure 1. Schematic diagram showing valve interconnections of prototype instrument. The arrows show the normally open pathway through the valves.*

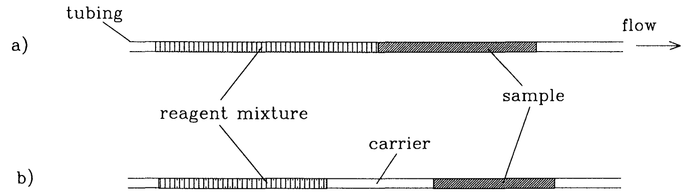

*Figure 2. Two of the reagent–carrier–sample mixing patterns which could be selected by the controlling software.*

In the present system, the sample loop volume was approximately 160 µl (obtained by using 25 cm of 0·035 in id, PTFE tubing), and the system used up to about 400 µl of reagent mixture (when operating in maximum sensitivity mode) and a total of 400 µl of sample (including the volume in the sample delivery tube) for each analysis. This latter volume could have been made smaller, although it compares favourably with the actual sample usage in other automatic analysis systems. Closure of valves 4 and 6 caused this sample containing sandwich to be passed through to the reaction tube (150 cm of 0·032 in id PTFE tubing – a volume of approximately 750 µl) and ultimately to the detector. Once the reaction mixture was in the reaction tube, valve 6 could be opened to stop the flow for a precisely controlled reaction time. As the reaction tube was maintained at a user-specified temperature, a wide variety of reaction times and temperatures could be employed to accommodate a range of reaction chemistries. After a timed reaction period valve 6 was opened and the reaction mixture passed through the detector system and to waste. For the reaction systems described below the flows were produced using a gas pressure of 20 psig which generated a liquid flow rate of approximately 5 ml min$^{-1}$ at the detector waste.

A particularly useful modification of these reaction procedures involves the measurement of the reaction product concentration in the tail end of the reaction mixture, as illustrated in figure 3. The reagent-sample pattern generated in the manifold was that illustrated in figure 2(*a*) (maximum sensitivity mode), and after passage through the reaction coil the profile of the reaction product concentration would have the appearance shown in figure 3. At the end of the reaction time valve 5 would be closed and the reaction mixture passed from the reaction coil. However, in this case valve 5 would be opened for a time period $t_d$ (which we still refer to as the dilution time) and the portion of the product profile to the left of $t_d$ would be passed to waste. Valve 5 would be closed and the remaining portion of the reaction mixture (to the right of $t_d$ in figure 3) would be passed

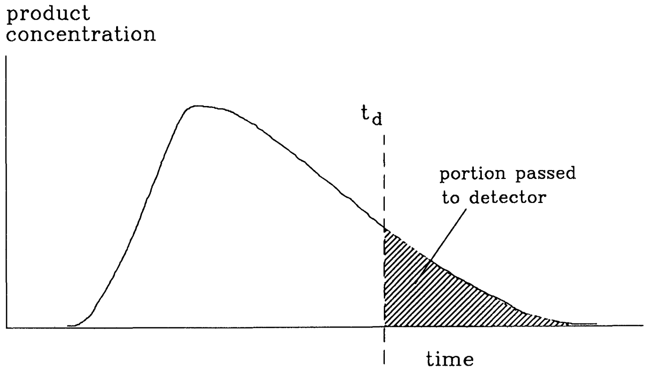

*Figure 3. Reaction product concentration profile on exit from the reaction tube, illustrating the point (td) at which product may be diverted to the detector so that only a diluted portion of the reaction mixture is used for peak height measurement.*

through the detector and the product concentration recorded. This approach was useful for high concentration samples, where either the product absorbance, or the depletion of reagent, would be very large and result in operation outside the linear range of normal operation. It also has an advantage over the in-line dilution technique (figure 2[*b*]), in that the refractive index effects observed in a reagent–carrier–sample pattern are greatly reduced.

The second modification incorporated in the instrument has been the inclusion of valve 16, which allows the sample to be diverted through a column during its passage to the reaction tube. In the present work, this column was filled with cadmium metal filings, which were then coated with copper. Passage of aqueous nitrate ion solutions through the column allowed the nitrate to be reduced to nitrite ion [3 and 4] (with an estimate 58% conversion efficiency over the $NO_3^-$ range 0–50 ppm), thus permitting the nitrite analysis reaction to be used for the measurement of nitrate. The column was fabricated from a Nylon, or Kel-F, rod drilled to provide a 25 mm pathway of 2 mm diameter to hold the cadmium filings, and provided with screw connectors to accept Altex fittings. The column could be reactivated by the passage of a few ml of an aqueous 0·1 M EDTA solution containing 0·1% $CuSO_4$.

The system's software allowed the user to define a procedure for specific analytical determinations. Each procedure consisted of a definition of the carrier bottle to be employed (see figure 1), the reagent bottle, or bottles, from which the reagents were obtained and the associated fill time, the reaction tube temperature, the wait time (*i.e.* the time the reaction mixture was held stationary in the reaction tube), the dilution time, $t_d$, and the detector's monitoring wavelength. The parameters used to convert from the detector's output signal to analyte concentration were determined during the calibration procedure and stored along with the procedure on disk. Two options are provided: linear least squares and interpolation, the latter being useful for those analyses which do not show linear calibration curves. The procedures also allowed for special activities, such as the regeneration of the cadmium reducing column, to be incorporated if required.

In normal operation the user loaded the required procedure from disk and was then given the choice of calibrating the procedure or carrying out analyses. Any number of replicate determinations could be specified, and procedures defined for the determination of up to five analytes sequentially from a given sample solution. The manual presentation of samples required the user to place the instrument's sampling tube into each sample bottle when prompted and press the front panel button when ready, although it is more likely that an instrument of this kind would be operated with an autosampler or by repeatedly sampling material from a single source (for example a process flow or effluent).

During analysis, the detector's output was illustrated on the computer display screen both graphically and digitally, and procedures allowed for graphics hard copy to be obtained on a chart recorder. The derived analyte concentration was normally printed on a printer along with statistical data when replicate determinations had been specified.

When the instrument was first used after power up, or a bottle refill, the connecting tubes were briefly flushed to expel air bubbles, and when the instrument was closed down the gas pressure within reagent bottles was released. Reagent bottles could contain sufficient reagent for 1000 determinations, so the instrument could operate for long periods without requiring manual intervention.

*Nitrite ion determination*

Nitrite ion determinations were carried out by reaction with a single reagent solution containing 10·0 g sulphanilamide, 0·8 g of N-naphthylamine ethylenediamine hydrochloride and 10·0 ml of concentrated hydrochloric acid per litre. The carrier was water containing 15 g $l^{-1}$ NaCl and 10 ml $l^{-1}$ concentrated HCl. For high sensitivity operation, the reaction coil was maintained at 50·0 ± 0·5 °C and a reaction time of 12 s was employed. The reaction mixture was then monitored at 545 nm. Figure 4 illustrates a typical calibration graph for nitrite standards (made from a stock solution containing 1000 ppm $NO_2^-$ as $NaNO_2$) covering the range 0–100 ppb $NO_2^-$.

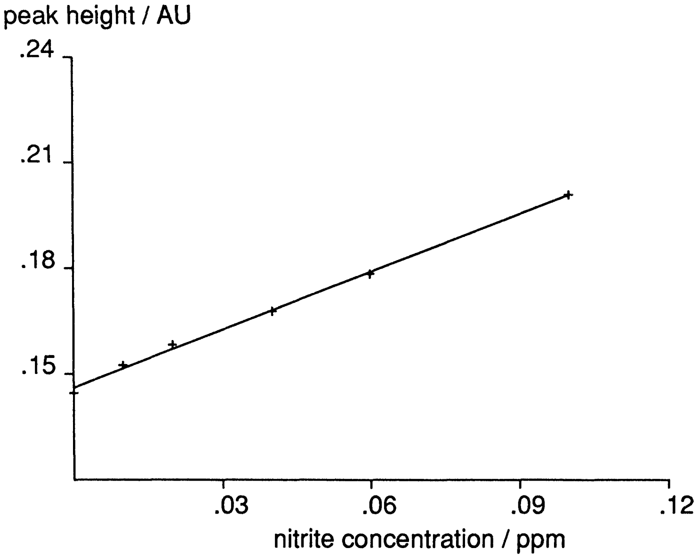

*Figure 4. Typical calibration curve obtained for standards of nitrite ion.*

*Nitrate ion determination*

Nitrate ion determinations were carried out by diverting the sample flow via the cadmium reducing column and determining the nitrite ion produced. The procedure for nitrate determination could include a nitrite determination, so that the actual nitrate level could be determined by difference. However, most of the trials reported in this work were carried out using drinking water supplies, and in these samples the nitrite level is so much lower than the nitrate level that such corrections are not really required. Furthermore, as the nitrate level in drinking waters is relatively high, the reduced nitrate determination may be carried out without stopping the flow in the reaction coil, and without the necessity to heat the reaction coil above ambient temperatures. Whether these conditions are employed, or the conditions are maintained the same as for the low level nitrite determinations of determined by the user's preference for the sequence of analysis, i.e. either analysing for nitrite and nitrate from each sample before proceeding to the next, or analysing a batch of samples for nitrate and then analysing the batch for nitrite. The latter course was chosen, with the nitrate determinations carried out at room temperature. To cope with the high levels of nitrate encountered in typical samples, a dilution time (see above) of 8 seconds was adopted, which was determined from the passage of copper sulphate solutions and gave rise to a peak height at the detector a factor of 10 smaller than the full reaction mixture. Figure 5 illustrates a typical calibration graph for nitrate ion standards (made from a stock solution containing 1000 ppm $NO_3^-$ as $NaNO_3$) covering the range 0–50 ppm $NO_3^-$.

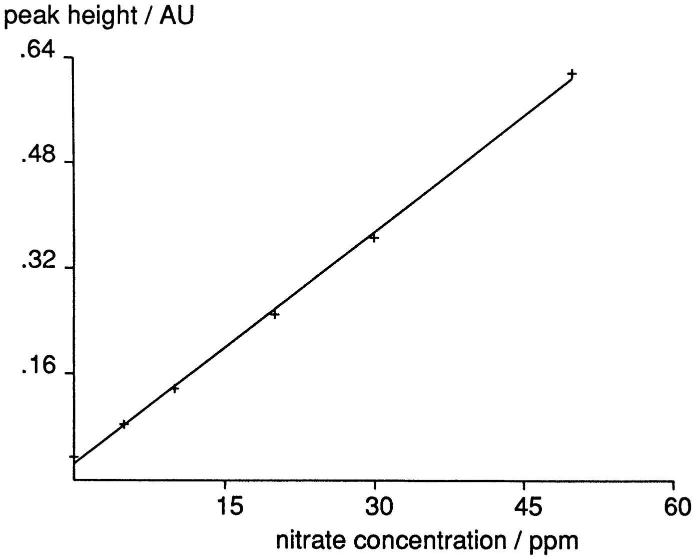

*Figure 5. Typical calibration curve obtained for standards of nitrate ion.*

*Ammonium ion determination*

Ammonium ion determinations were carried out using the Berthelot reaction catalysed by sodium nitroprusside. Three reagent solutions were employed: 0·5% phenol (w/v) containing 0·5% (w/v) sodium nitroprusside, 0·15% sodium hypochlorite solution, and a masking solution of 0·35 M NaOH containing 1·5% (w/v) EDTA (disodium salt). The carrier was aqueous 0·05 M NaOH containing 15 g $l^{-1}$ NaCl. The reaction coil heater was operated at 50·0 °C, and a reaction time of 12 s was employed. The mixture's absorbance was monitored at 690 nm. Standard solutions were prepared from a stock solution containing 1000 ppm $NH_4^+$ as $(NH_4)_2SO_4$, and a typical calibration graph covering the range 0–120 ppb $NH_4^+$ is shown in figure 6.

The procedures for these analyses and the typical sample throughputs are summarized in table 1.

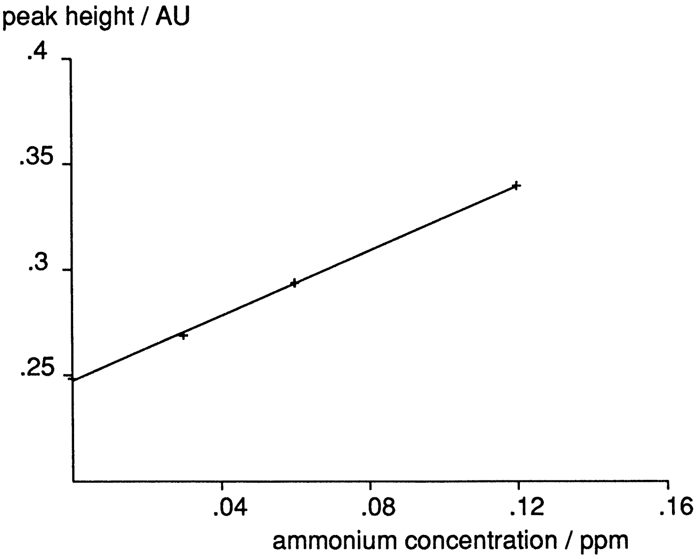

*Figure 6. Typical calibration curve obtained for standards of ammonium ion.*

*Table 1. Parameters associated with the nitrogen analysis procedures.*

| Parameter | Ammonium | Nitrate | Nitrite | Units |
|---|---|---|---|---|
| Dilution factor | 1 | 10 | 1 | |
| Wait time | 12 | 0 | 12 | s |
| Fill time | 6·5 | 5·0 | 5·0 | s |
| Wash time | 25 | 17 | 25 | s |
| Reaction temperature | 50 | ambient | 50 | C |
| Sample throughput | 70 | 90 | 70 | /hour |

### Results and discussion

Trials of the system were carried out using eight drinking water samples, some of which were collected from various parts of the UK and others of which were samples kindly provided by the Laboratory of the Government Chemist (LGC). Table 2 shows the results obtained for nitrate

*Table 2. Results for nitrate ion determinations.*

| Sample No. | Present result (ppm) | Reference method result (ppm) |
|---|---|---|
| 1 | 20·0 ± 0·2 | 19·9[a] |
| 2 | 3·6 | 3·6[a] |
| 3 | 35·6 | 33·2[a] |
| 4 | 2·5 | 2·8[a] |
| 5 | 16·0 | 15·8[b] |
| 6 | 19·7 | 20·8[b] |
| 7 | 24·6 | 25·7[b] |
| 8 | 6·7 | 5·7[b] |

*Notes*
(a) Official method reference [8].
(b) Dionex ion chromatograph.

determinations, compared with independent results obtained using either the official method [8] (reaction with sulphosalicyclic acid to form a yellow colour), or by measurement on a Dionex ion chromatograph at the LGC.

The authors did not have access to independent results for the determination of nitrite and ammonium for the levels present in the samples, so results are presented for the determination of these species in unadulterated samples and for the same samples spiked with a known additional concentration of each ion. This leaves the host matrix relatively undisturbed, but allows some indication of the reliability of the results to be derived. The results for nitrite and ammonium determinations are collected in table 3.

*Table 3. Results for nitrite and ammonium determinations.*

*Nitrite results*

| Sample No. | Unspiked (ppb) | Spiked to (ppb) | Spiked result (ppb) |
|---|---|---|---|
| 1 | 143 | 145 | 147 |
| 2 | 95 | 120 | 123 |
| 3 | 75 | 81 | 83 |
| 4 | 57 | 107 | 106 |
| 5 | 47 | 80 | 83 |
| 6 | 4 | 22 | 19 |
| 7 | 0 | 62 | 58 |
| 8 | 4 | 11 | 9 |

*Ammonium results*

| Sample No. | Unspiked (ppb) | Spiked to (ppb) | Spiked result (ppb) |
|---|---|---|---|
| 1 | 43 | 72 | 68 |
| 2 | 20 | 60 | 59 |
| 3 | 0 | 15 | 13 |
| 4 | 15 | 148 | 153 |
| 5 | 15 | 88 | 93 |
| 6 | 15 | 58 | 61 |
| 7 | 28 | 55 | 55 |
| 8 | 40 | 205 | 200 |

From a large number of calibration graphs of the kind illustrated in figures 4, 5 and 6, the sensitivities and precision of the procedures are estimated, and the corresponding limits of detection (evaluated as twice the standard deviation of a blank divided by the sensitivity), are as summarized in table 4. The sensitivity for the ammonium determination significantly higher than reported previously [2], as a result of electronic improvements which have been made to the variable wavelength detector and the new 2·5 cm path length flow cell incorporated.

The results indicate that the instrument is capable of a precision, sensitivity, reproducibility and sample throughput at least as good as those obtained previously

*Table 4. Derived characteristics of the analytical procedures.*

| Analyte | Sensitivity AU/ppm | Precision AU (10 readings) | LOD |
|---|---|---|---|
| $NO_2^-$ | 0·6936 | 0·0015 | 4 ppb (1 ppb as N) |
| $NO_3^{-a}$ | 0·018 | 0·0022 | 0·22 ppm (0·05 ppm as N) |
| $NO_3^{-b}$ | 0·1229 | 0·0018 | 0·03 ppm (7 ppb as N) |
| $NH_4^+$ | 0·6395 | 0·0020 | 5 ppb |

*Notes*

(a) Using the dilution factor of 10 as described above.
(b) Using a dilution factor of 1, 10 s reaction time at 50 °C.

for separate ammonium and nitrate/nitrite determinations. Thus the system used as described can determine ammonium ion down to 5 ppb at 70 × 0·3 ml samples per hour. (An upper limit has not been determined because the flexibility of the slicing and in-line dilution [2] techniques allow this to be varied considerably.) Slanina *et al.* used the Berthelot reaction for the determination of ammonium ion in rain waters and were able to operate over the range 0·2–20 ppm handling 18–35 × 0·5 ml samples per hour. Krug *et al.* [9] used Nessler's reagent for the turbimetric determination of ammonia in natural water, finding a linear calibration over the range 0·7–7·0 ppm $NH_4^+$ and achieving a throughput of 120 samples per hour with relatively small sample sizes (30 μl). The present system can handle nitrate determinations down to 30 ppb at a throughput of 70 samples per hour, or down to 220 ppb at a throughput of 90 samples per hour. The performance of the instrument could almost certainly be improved for nitrate through optimizing the reduction system, although there is currently little demand for this.) The procedure described has been used for the analysis of nitrate in ground waters and biological growth media for concentrations up to 500 ppm $NO_3^-$. For nitrite the instrument may be used at levels down to 4 ppb, again with a throughput of 70 samples per hour. Madsen [10] carried out nitrate analyses and achieved a range of 1–10 ppm with a throughput of 40 1 ml samples per hour, and Nakashima *et al.* [11] achieved a range of 0·7–100 ppb for nitrite ion using 30 × 0·65 ml samples per hour (although using a different reaction from the one employed in this work). A variety of analyte ranges and sample throughputs for these analytes have been summarized [12].

However, the principal attraction of the present instrument is that it can be programmed to carry out all three of the analyses described, sequentially, either from each sample in turn or with each analyte being determined from a group of samples before the sample group is re-analysed for the next analyte. This allows a single sample, for example, to be analysed for the three analytes within minutes of the instrument being switched on. This flexibility can be valuable in a situation where a rapid result for several analytes is required from a single sample, such as in medical diagnosis. Furthermore, the concentration of the analytes may be dramatically different because the procedure for each analysis may be programmed to incorporate an independent dilution factor as described above (or to use the in-line dilution technique described previously [2]).

### Acknowledgements

Some aspects of this work were performed with the aid of support from the Royal Society. The authors are also grateful for the advice of Dr R. Newton of Biotech Instruments and for the co-operation of Dr R. Moxon of the Laboratory of the Government Chemist in providing samples and the results of reference analyses. CP was on leave from Universidade Estadual de Campinas - SP, Brazil. DJM-L is a Royal Society Research Fellow in the Physical Sciences.

Commercial versions of the instrument described are available from Biotech Instruments Ltd, 183 Camford Way, Luton LU3 3AN, UK.

### References

1. Malcolme-Lawes, D. J., Milligan, G. A., and Newton, R., *Journal of Automatic Chemistry*, **9** (1987), 179.
2. Malcolme-Lawes, D. J. and Pasquini, C., *Journal of Automatic Chemistry* (in press).
3. Anderson, L., *Analytica Chimica Acta*, **110** (1979), 123.
4. Gine, M. F., Bergamin, H. F., Zagatto, E. A. G. and Reis, B. F., *Analytica Chimica Acta*, **114** (1980), 191.
5. van Staden, J. F., *Analytical Chimica Acta*, **138** (1980), 403.
6. Zagatto, E. A. G., Jacintho, A. O., Mortatti, J. and Bergamin, F. H., *Analytica Chim Acta*, **120** (1980), 399.
7. Slanina, J., Bakker, F., Bruyn-Hes, A. and Mols, J. J., *Analytica Chimica Acta*, **113** (1980), 331.
8. *Oxidised Nitrogen in Waters*, 1981, (HMSO, London, 1982), 31.
9. Krug, F. J., Ruzicka, J., and Hansen, E. H., *Analyst*, **104** (1979), 47.
10. Madsen, B. C., *Analytica Chimica Acta*, **124** (1981), 437.
11. S. Nakashima, M. Yagi, Takahashi, A., and Toei, K., *Analytica Chimica Acta*, **155**, (1983), 263.
12. Valcarcel, M. and Luque de Castro, M. D., *Flow Injection Analysis Principles and Applications* (Ellis Horwood Ltd, Chichester, 1987).

### AUTOMATED DETERMINATION OF MERCURY ANALYSIS AT LOW LEVELS

Amongst the various pollutants receiving attention in the UK water authorities is mercury. Legislation to prevent contamination of rivers and raw and potable waters is being introduced which puts pressure on analysis to measure this determinant accurately at very low levels, certainly below 0·1 ppb. The generally accepted method involves the evolution of mercury as a vapour and determination by atomic absorption techniques using long path-length cells. The new legislation stretches the limits of this approach and the inherent memory effects of the cells used prevents analytical rates achieving the necessary speeds.

P S Analytical, in association with the Yorkshire Water Authority, has developed a fully automated system based on atomic fluorescence. The system comprises a large volume autosampler; a vapour generator, specifically tailored for mercury analysis; and a fluorescence spectrometer. These are interfaced to an IBM compatible PC running PSA TouchStone Software. All the instruments are controlled by the PC and the data collected is analysed and reported by the software. The vapour generator produces a reliable means of providing the mercury vapour and the PSA developments have resulted in an efficient transfer and measurement of the mercury. A major advantage of the interface is that the rate of rise and fall on the signal is rapid, enabling fast analysis at extremely low levels. Range gain changes consistent with levels in the range 0–20, 0–1 and 0–0·1 ppb are easily attainable, with detection levels of approximately 25 parts per trillion being obtained by customer sites.

Coupling the P S Analytical mercury measurement system with an on-line digestion approach, or a batch Prolabo A300 Microwave Digestion system, enable the total inorganic/organic mercury to be measured.

*Further details from: P S Analytical Ltd, Arthur House, Far North Building, Cray Avenue, Orpington, Kent BR5 3TR, UK.*

